# Medical Association of Georgia

**Published:** 1883-05-20

**Authors:** 


### *MEDICAL ASSOCIATION OF GEORGIA.*

It so happened that neither of the editors of this journal could be present at the late meeting of the Medical Association of the State. An account of the proceedings of the body will be found in another part of our journal, kindly furnished us by a member who was present. The account of the meeting, apart from its social features, is not specially flattering. There seems to have been too much haste, and a premature adjournment.

It is a matter of regret that comparatively few of our best men and ablest writers attend the meetings, and hence the interest in the Association is declining, and the volume of transactions growing smaller with each succeeding year. It would seem to be the tendency of State Societies to fall under the control of cliques or rings, who make the appointments and distribute the honors in their own interest, and the impression prevails that the Georgia Association is not wholly free from this charge. But whatever may be said upon this point, we are pleased to state that in the choice of Dr. Calhoun, as President for the ensuing year, we have a man eminently worthy of the position; and we mean no disparagement to his predecessors in expressing the belief that his administration will be wise and *impartial* and directed to the best interest of the profession.

